# Pressure Drop in Tortuosity/Kinking of the Internal Carotid Artery: Simulation and Clinical Investigation

**DOI:** 10.1155/2016/2428970

**Published:** 2016-04-18

**Authors:** Lijun Wang, Feng Zhao, Daming Wang, Shen Hu, Jiachun Liu, Zhilun Zhou, Jun Lu, Peng Qi, Shiying Song

**Affiliations:** ^1^Department of Neurosurgery, Beijing Hospital, Beijing 100730, China; ^2^Key Laboratory for Biomechanics and Mechanobiology of the Ministry of Education, School of Biological Science and Medical Engineering, Beihang University, Beijing 100191, China

## Abstract

*Background*. Whether carotid tortuosity/kinking of the internal carotid artery leads to cerebral ischemia remains unclear. There is very little research about the hemodynamic variation induced by carotid tortuosity/kinking in the literature. The objective of this study was to research the blood pressure changes induced by carotid tortuosity/kinking.* Methods*. We first created a geometric model of carotid tortuosity/kinking. Based on hemodynamic boundary conditions, the hemodynamics of carotid tortuosity and kinking were studied via a finite element simulation. Then, an in vitro system was built to validate the numerical simulation results. The mean arterial pressure changes before and after carotid kinking were measured using pressure sensors in 12 patients with carotid kinking.* Results*. Numerical simulation revealed that the pressure drops increased with increases in the kinking angles. Clinical tests and in vitro experiments confirmed the numerical simulation results.* Conclusions*. Carotid kinking leads to blood pressure reduction. In certain conditions, kinking may affect the cerebral blood supply and be associated with cerebral ischemia.

## 1. Introduction

The correlation between tortuosity/kinking of the internal carotid artery and the occurrence of cerebral ischemia or ischemic stroke has long been a matter of dispute. Tortuosity includes the elongation, redundancy, undulation, and S-shaped curvature of the internal carotid artery. Kinking specifically refers to the situation in which the 2D projection of the inflection segments of the internal carotid artery exhibits a sharp angle of <90 degrees [1]. Perdue suggested that vascular elongation and tortuosity are benign [2]. Weibel and Fields demonstrated that carotid kinking causes luminal stenosis and probably cerebral ischemia only when atherosclerosis occurs [1]. On the other hand, a thin wall with the enlarged lumen diameter of the internal carotid artery kinked segment was demonstrated [3]. The correlation between carotid kinking and the occurrence of cerebral ischemia or ischemic stroke has been increasingly supported since 1951. Symptoms have been alleviated or disappeared following surgical therapy in many symptomatic patients with carotid kinking [4]. However, there are only case reports in the literature supporting this finding, and no prospective research on the hemodynamic variation induced by tortuosity and kinking of the internal carotid artery has been conducted [5]. In a severe carotid kinking dog model, the blood flow at the distal end was lower compared with the proximal end [6]. However, whether luminal stenosis or obstruction developed in the animal model was uncertain due to the lack of a credible imaging study. Moreover, the results derived from animal experiments cannot be easily extrapolated to humans due to the heterogeneity between the human and animal tortuous carotid artery. The aim of this study was to assess the blood pressure changes due to carotid kinking and tortuosity, which are key factors that affect the brain's blood supply. How carotid kinking at different kinking angles influences blood pressure variation was studied using numerical simulation, an in vitro experiment, and a clinical investigation. The effects of carotid kinking on the brain blood supply were studied, and the results could help to provide a rationale for surgical intervention.

## 2. Materials and Methods

### 2.1. Patient Selection and Clinical Data

Twelve patients with carotid kinking who underwent digital subtraction angiography (DSA) for cerebrovascular diseases at our hospital were selected based on the angle of kinking between December 2005 and May 2012. DSA was used to confirm the presence of carotid kinking without medium or severe luminal stenosis in the ipsilateral carotid artery. The twelve patients (seven males and five females) were aged between 41 and 76 (62.6 ± 12.8) years. Among the twelve patients, four were diagnosed with coronary heart disease, five with diabetes, four with hypertension, and seven with hyperlipidemia. Informed consent was obtained from all of the patients, and the study was approved by the hospital's ethics committee.

#### 2.1.1. Numerical Simulation

**Figure 1:**
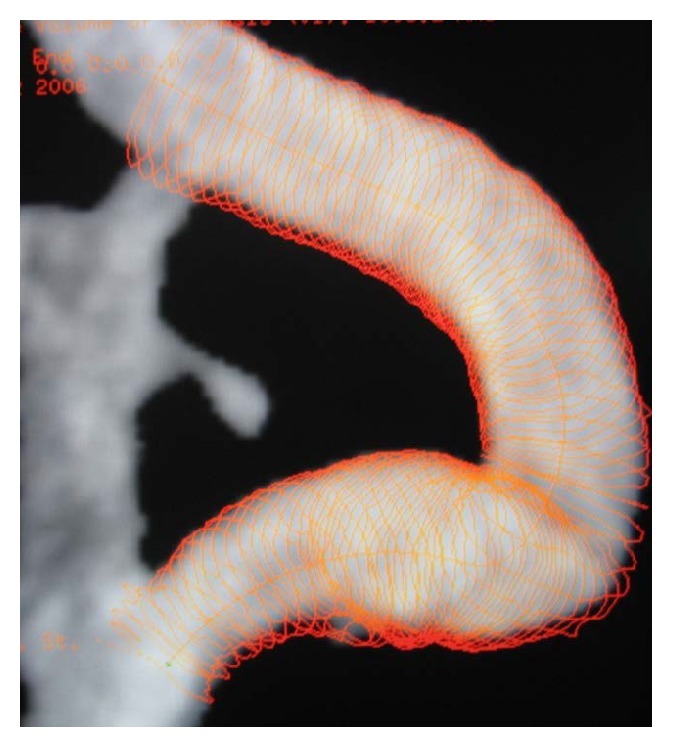
Digital subtraction angiography (DSA) illustrating carotid kinking.

**Figure 2:**
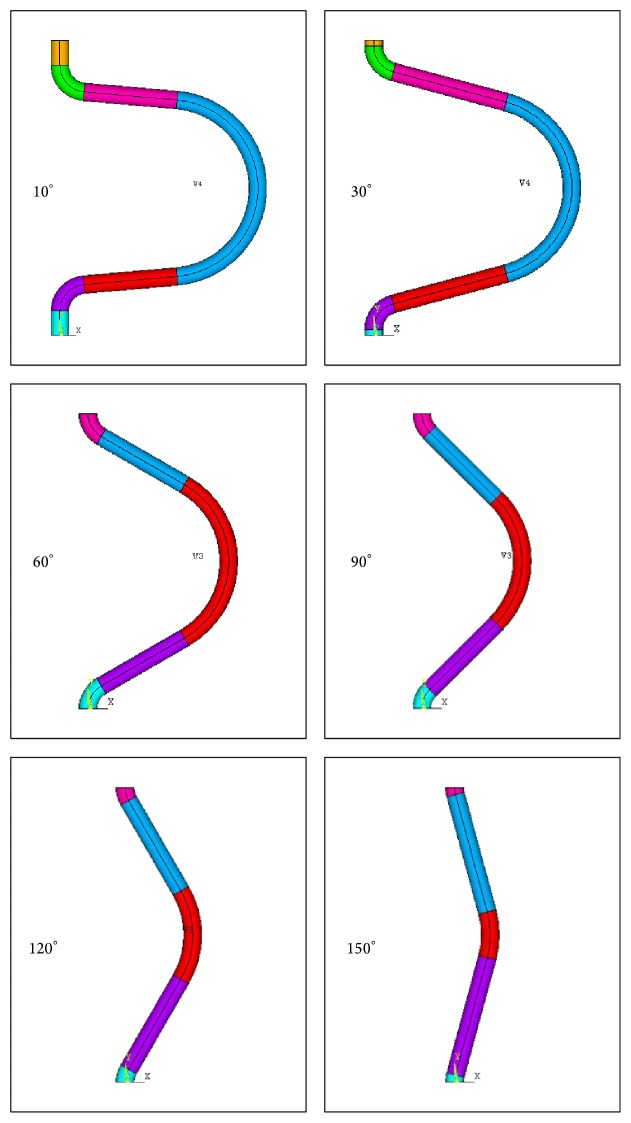
Geometric model of carotid kinking and tortuosity at different angle.

Based on the normal physiological parameters of the internal carotid artery, a geometric model of a tortuous carotid artery was built (Figures 1 and 2). The simplified assumptions included the following: (1) the cross section of the vessels was circular; (2) the blood was modeled as a homogenous, isotropic, and incompressible Newtonian fluid; (3) the motion of the blood in the artery was a steady laminar flow that satisfied the Navier-Stokes equation; and (4) the vascular wall was a rigid wall without viscoelasticity under nonslip conditions. A 3D geometric model of carotid kinking was built with the software ANSYS FLOTRAN. Numerical simulation was conducted with FLOTRAN CFD in ANSYS 10.0. Then, 3D FLUID142 elements were used as finite elements and divided by an automatic mesh method. After meshing, the total number of elements was 50000 with 10000 nodes. The walls were under nonslip rigid boundary conditions, and the outlet pressure was set at 0. During the solving process, the control conditions were set as follows: (1) the total number of iterations was 200, and (2) the converging precision of both velocity and pressure was 0.00001.

### 2.2. In Vitro Flow Simulation Experiment

**Figure 3:**
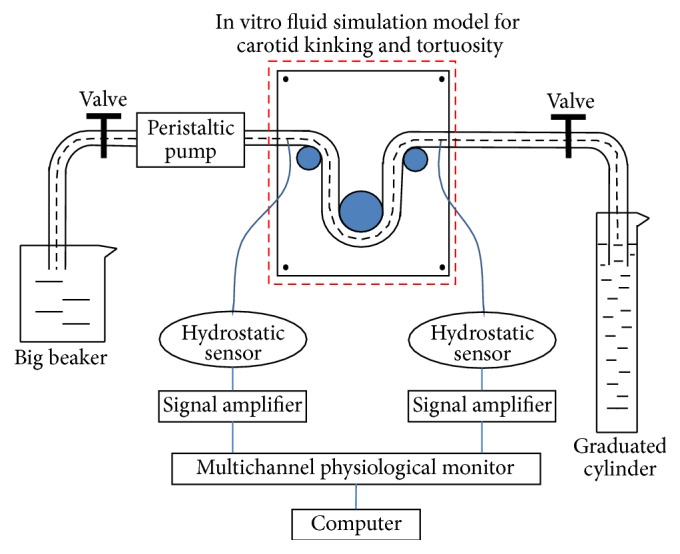
Diagram of homemade in vitro flow system for carotid kinking and tortuosity.

The homemade in vitro flow system for carotid kinking included 4000 mL beakers, a BT00-600M peristaltic pump (Longer China), silica gel tubes, disks, a Mikro-tip catheter with a pressure sensor (AD instruments), and a computer and a 1000 mL dosimeter. The signals obtained by the pressure sensor were amplified, collected with an ML870 Power Lab 8/30 data acquisition system, and then transferred to the computer (Figure 3).

In the experimental system, the human carotid artery was simulated using a silicon tube with water as the fluid medium. Water flowed through the silicone tubes at a constant rate via a peristaltic pump. Water flowed from a beaker into a peristaltic pump and then through the carotid kinking model into a dosimeter. The average flow velocity was measured via the dosimeter. The pressures at the different observation points were measured via a fluid pressure signal acquisition system composed of a pressure sensor, a signal amplifier, a multichannel physiological tester, and a computer. Carotid kinking with different kinking angles was created by a disc. Five sets of silicon tubes were measured. The values are expressed as the means ± the standard deviations.

### 2.3. Carotid Arterial Blood Pressure Measurements in the Patients

The carotid kinking was first confirmed via routine DSA in all patients. Then, the kinking angles were measured by rotational angiography using a protractor according to the Metz's method. The length of the kinking vessel was measured via the image processing system of an Advantx LCN+ (GE Medical Systems, USA) or PHILIPS FD 20/20. The blood pressure along the kinking carotid artery was measured as follows. The head of the 6F ENVOY guiding catheter (Cordis, USA) was placed into the initial part of the internal carotid artery. Following the road map, an Echenlon-14 microcatheter (EV3, USA) was passed through the proximal, kinking, and distal sites of the internal carotid artery under the guidance of a microwire. A pressure sensor (Abbott Critical Care Systems, USA) was connected to the tail of the microcatheter. The blood pressures at the proximal, kinking, and distal sites of the carotid artery were measured with a Dash 2000 multifunctional monitor (GE, USA). The measurements were conducted at a constant heart rate. The results were taken as the averages of three mean arterial pressure measurements after stabilization. For two patients with the same kinking angle, the final result was the average of their blood pressure readings.

## 3. Results

### 3.1. Numerical Simulation and In Vitro Experiment

**Figure 4:**
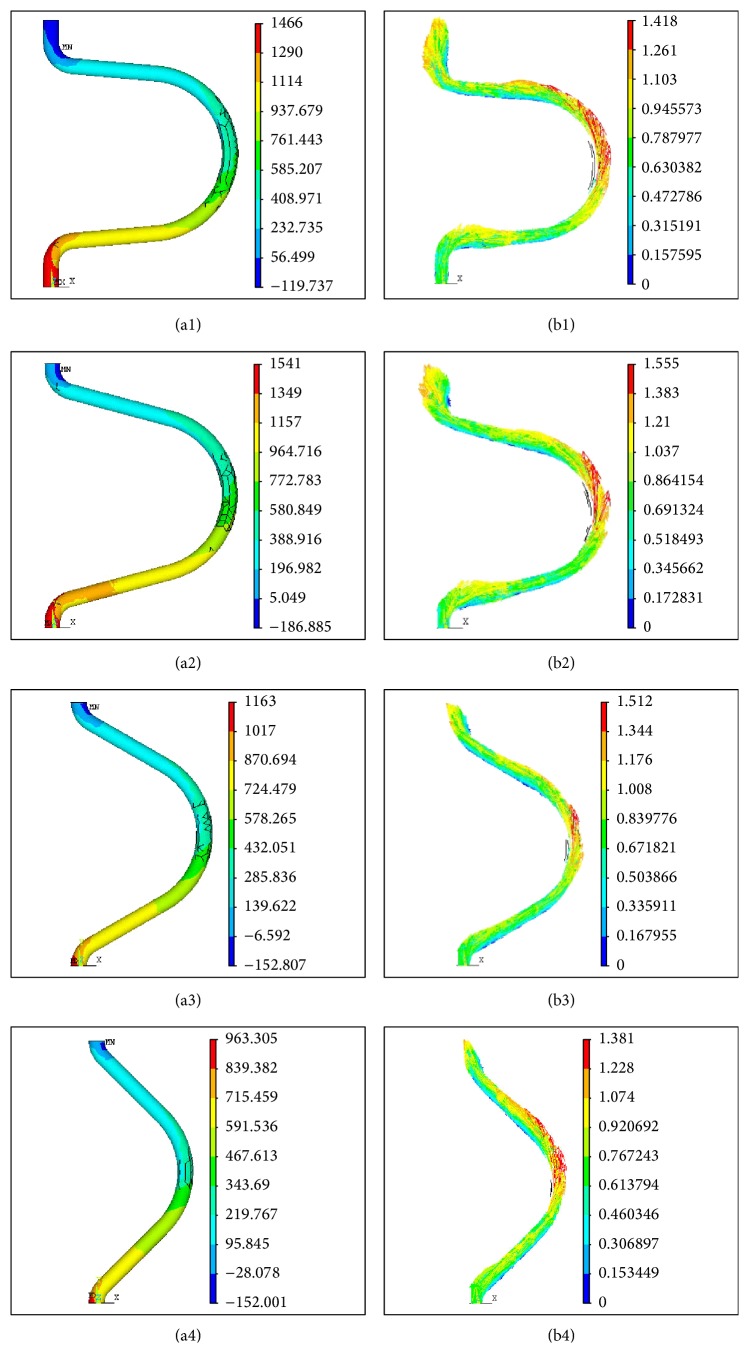
The results of the carotid kinking numerical simulation. (a1)–(a4) are the pressure distributions at the kinking angles of 10°, 30°, 60°, and 90°, respectively. (b1)–(b4) are the velocity distributions at the kinking angles of 10°, 30°, 60°, and 90°, respectively.

The numerical simulation revealed that the kinking angles dramatically changed the hydrokinetic indices, such as the blood pressure and flow field, and the flow velocity was particularly dramatically altered at the kinking part. In other words, the smaller the kinking angle, the greater the vascular distortion degree, which thus induced more significant blood pressure and flow field changes at the kinking part.  Figure 4 illustrates the results of the carotid kinking numerical simulation. (a1)–(a4) illustrate the pressure distribution, and (b1)–(b4) illustrate the flow field at kinking angles of 10°–90° (Figure 4).

With constant blood flow at the inlet, the blood flow pressure drop increased linearly (correlation coefficient *R* ^2^ = 0.9819) with decreases in the kinking angle. The pressure drop was the smallest at the kinking angle of 180°, that is, the straight tube, and was the largest at 30° by approximately 4-fold greater than that in the straight tube. However, a reversal occurred between the kinking angles of 20° and 30°; the increasing kinking degree led to a smaller pressure drop, which was still greater than that in the straight tube.

This was confirmed in the in vitro experimental system.  Table 1 presents the pressure drops at different tortuosity angles in the in vitro experiment system. When the flow rate was constant, the pressure drop decreased with increasing kinking angle, but the pressure drop increased with the increased kinking angle when the kinking angle increased from 20° to 30°. The value of pressure drop in the in vitro experiment was remarkable, but the same trend was observed in the theoretical calculations.

### 3.2. Clinical Tests

Twelve carotid kinking patients with different angles underwent mean arterial pressure measurements. The kinking angles were below 30° in four cases, between 30° and 60° in 5 cases, and two patients exhibited the same kinking angle of 45°. The final result was the average of their blood pressure readings, and 3 cases were between 60° and 90° (Table 2).

**Figure 5:**
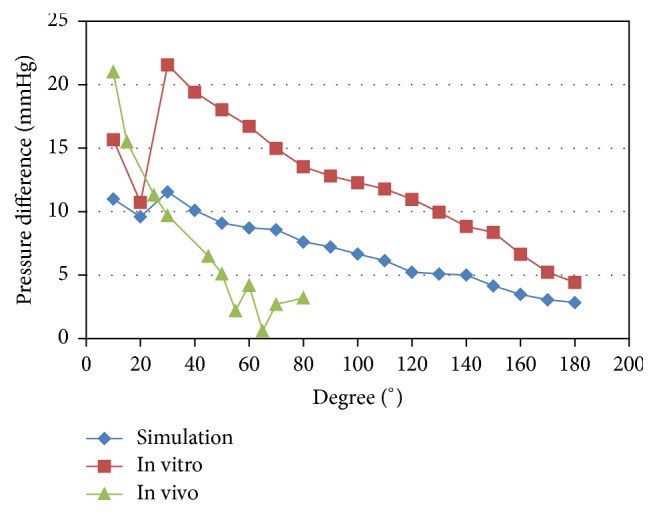
Pressure differences in the numerical simulation, in vitro flow system, and clinical tests.

The clinical findings are illustrated in Figure 5. The kinking angle variations elicited remarkable pressure drop increases when they were less than 30°, and these increases were roughly linear; however, when the kinking angles were greater than 30°, the pressure drop variations were not as obvious, and the distribution was nonlinear.

The pressure drop increased with decreased kinking angle in the clinical tests, and the same trend in the changes was observed in the in vitro experiments and numerical simulation results. However, the development of the reversal of greater pressure drops with increased kinking angles from 20° to 30° was not observed in the clinical measurements.

## 4. Discussion

The regulation of cerebral blood flow is affected by many factors. The key determinants are the arterial blood pressure, the venous pressure difference, and the cerebral vascular resistance. The regional cerebral blood flow can be directly measured with SPECT, CTP, and PET. It is also common to use hemodynamic parameters, such as blood pressure and the length and resistance of the artery, to calculate the cerebral blood flow indirectly in many cases.

We chose MAP as the object of the research based on the assumption that the intracranial pressure and cerebral vascular resistance were constant. A decline in MAP may lead to a decrease in the cerebral blood flow.

Previous experiments with carotid kinking in animals have reported drops in blood flow [5], but there is no convincing imaging evidence of carotid kinking. There is no previous theoretical research about the blood pressure variations induced by carotid kinking or numerical simulations of carotid kinking. In our study, a numerical simulation, in vitro experiment, and intra-arterial blood pressure measurements were used to analyze the blood pressure variations induced by carotid kinking and tortuosity.

The results revealed that under a constant flow rate, the pressure drop before and after the kinking and tortuosity increased with decreases in the kinking angle, and these results were further confirmed in the in vitro experiments. Therefore, from the biomechanical perspective, it was confirmed that carotid kinking can induce a blood pressure drop. Because kinking causes more obvious blood pressure drops and slight tortuosity, kinking patients were selected and clinically tested.

Although the relationship between carotid kinking and ischemic stroke was first proposed in 1951 [7], the relationship between carotid kinking and ischemic cerebrovascular diseases has been extensively debated [8]. Carotid kinking is observed in 4–25% of patients with symptomatic ischemic cerebrovascular diseases [9]. However, Oliviero et al. did not find an increasing rate of ischemic stroke in patients with carotid kinking compared to normal controls after 7 years of follow-up [10]. This controversy was partly due to different definitions of carotid kinking and/or different examination methods. Moreover, because cerebral ischemia can be influenced by many factors, it is very difficult to clarify the relationships of single factors with cerebral ischemia. Carotid kinking patients usually have many complicating diseases, and clinical studies alone cannot provide definitive evidence. Therefore, basic research and clinical examinations of hemodynamics are both very necessary for studies of carotid kinking. In this paper, a geometric model was created, and in vitro experiments were then set up to study the hemodynamic changes in carotid kinking. Finally, the carotid artery blood pressure changes were measured in 12 patients with carotid kinking without serious stenosis.

The numerical simulation and in vitro experiment results indicated that carotid kinking led to decreases in blood pressure. However, these results were greatly different from those in the human body. With the progress and development of interventional materials and technology, pressure guide wires and intravascular ultrasound have been applied in patients with coronary heart disease. The wide applications of microcatheters and microwires have made safe invasive hemodynamic monitoring possible in human blood vessels. In this study, endovascular microcatheter technology was used to measure the arterial pressures in carotid kinking patients. The results revealed that carotid kinking induced decreases in the mean arterial pressure (MAP). In our clinical tests, severe kinking induced an average decrease of 15.5% (15.5/99.7) in arterial pressure. Although this drop was not remarkable, it validated the notion that carotid kinking could induce a blood pressure decrease in humans.

A difference between the simulation and human data was the slope of the pressure change with decreasing kinking angle. The pressure drop changed gradually with angle variation in the numerical simulation and in the in vitro experiment but decreased rapidly when the kinking angle was less than 45° in the clinical measurements. This discrepancy can be attributed, at least in part, to the following two differences between the in vitro/in silico and in vivo studies. First, in the numerical simulation and the in vitro experiment, the medium was water, which is a Newtonian fluid, whereas, in the in vivo study, the medium was blood, which is a non-Newtonian fluid. The viscosity of the blood is greater than that of water, and it also varies with the flow shear rate. Second, in the numerical simulation and the in vitro experiment, the tubes were rigid, whereas the carotid arteries are elastic. Both blood viscosity and carotid artery elasticity can contribute to the self-regulatory mechanism in the human body. Thus, pressures decrease only slightly within the compensatory range of self-regulation but change dramatically beyond the range of self-regulation.

Another discrepancy was the reversal development. Greater pressure drops were observed with increased kinking angles from 20° to 30° in both the in vitro experience and the simulations. The reversal development was not observed in the clinical measurements. The possible explanations may be that we set the length of the carotid artery at 10 cm according to physiological parameters in the experimental measurements and theoretical calculations. When the tortuosity angle reached 30° or below, the length exceeded 10 cm. The extra part was set as another mild tortuosity. However, in vivo, extreme tortuosity of the carotid artery was accompanied by excessive elongation, which might form another tortuosity or lead to luminal stenosis. Therefore, the pressure drop continues to increase as the tortuosity is aggravated. This discrepancy requires further studies to verify. Because the interval between 20° and 30° was a small portion of the tortuosity angles, as illustrated in Figure 5, the trend in the angle-pressure drop graph was still obvious.

Monitoring during surgical treatment requires carotid artery puncture, which is highly invasive and dangerous and cannot be performed preoperatively. Occasionally, such monitoring cannot be performed when the artery is in a high position. Intravascular microcatheter technology with high credibility and low invasion can be conducted during routine preoperative DSA. The clinical measurements in this study proved that an intravascular microcatheter was a feasible preoperative assessment method.

Although carotid kinking can induce blood pressure decreases, the induced pressure drop was only 21 mmHg (1 mmHg = 0.133 kPa) even with the most severe carotid kinking (kinking angle < 30°). Generally, an individual ensures adequate cerebral blood flow via the self-regulatory mechanism even when there is a slight blood pressure drop. However, the brain's self-regulatory mechanism is weakened in old people, especially those with hypertension, diabetes, or arteriosclerosis. Cerebral ischemia will occur when the self-regulatory mechanism cannot compensate for the blood flow drop induced by carotid kinking, and this may explain why only a proportion of carotid kinking patients develop cerebral ischemia symptoms.

In addition to hemodynamic variations, carotid kinking may also induce luminal stenosis. Doppler ultrasound examinations have revealed that among 199 patients, the kinking-induced vascular luminal stenosis degree exceeded 60%, and the peak systolic blood flow velocity of the involved segments exceeded 150 cm/s [11]. To simplify our model, no stenosis or other kinking was considered in the numerical simulation or the ex vivo experiment. This may be the reason that reversals occurred between the kinking angles of 20° and 30° in the numerical simulation and the in vitro experiment but differed in vivo. In the numerical simulation and the in vitro experiment, the horizontal range of the kinking carotid was set at 10 cm according to the normal neck circumference range of 30–40 cm because the kinking range could not extend in an unrestricted transverse manner in the patients, but in the numerical simulation and the in vitro experiment, when the kinking angle was less than 30°, the kinking range exceeded 10 cm. We let the assumed carotid and silica gel tubes turn right or left without stenosis or tortuosity, which may have slightly affected the pressure drop but differed from the real situation. Indeed, the most likely changes in patients may be vessel wall collapse and luminal stenosis or additional kinking and tortuosity.

We must clarify that this study has some limitations because a few assumptions were made to simplify the simulation. First, the idealized geometry model did not represent the real elastic behavior of real vessel walls. The applied silicon does not possess the same elastic properties indicated by the compliance values defined for arteries. Second, atherosclerosis plaque formation and the consequent hemodynamic influence were not investigated in this paper. Third, we used a Newtonian working fluid in our study, but blood is a non-Newtonian fluid. However, in vessels larger than 0.5 mm in diameter, blood is considered to behave as a Newtonian fluid.

## 5. Conclusions

Carotid kinking can cause a drop in blood pressure. The blood pressure drop increases with decreases in the kinking angle, but the blood pressure drop is limited. In most cases, the drop cannot lead to cerebral ischemia because of the compensation of the self-regulatory mechanism in the cerebral blood supply. When the self-regulatory mechanism is weakened or decompensation occurs due to factors such as atherosclerosis, hypertension, diabetes, or old age, carotid kinking may cause cerebral ischemia.

## Figures and Tables

**Table 1:** Pressure differences and fluxes in the in vitro carotid kinking experiment.

Angle (°)	Pressure difference (mean ± SD, mmH_2_O)	Volume flux (mean ± SD, mL/min)
10	156.7 ± 16.7	300.2 ± 1.8
20	107.34 ± 12.3	298.4 ± 1.5
30	215.61 ± 24.5	295.8 ± 1.2
40	194.17 ± 20.6	301.3 ± 0.8
50	180.27 ± 15.4	298.6 ± 1.1
60	167.25 ± 13.7	298.2 ± 1.2
70	149.85 ± 10.8	298.4 ± 1.6
80	135.31 ± 18.4	298.6 ± 0.9
90	128.12 ± 9.8	299.1 ± 1.4
100	122.82 ± 12.1	300.2 ± 1.4
110	117.8 ± 28.9	298.9 ± 1.7
120	109.72 ± 10.3	300.1 ± 1.6
130	99.56 ± 8.6	300.2 ± 1.8
140	88.41 ± 7.4	297.4 ± 2.1
150	83.67 ± 8.2	300.1 ± 0.9
160	66.57 ± 5.6	300.3 ± 1.8
170	52.34 ± 4.5	299.6 ± 1.3
180	44.29 ± 3.8	299.8 ± 1.2

**Table 2:** Mean arterial blood pressures in carotid kinking.

Patient	Angle	Length (cm)	^*∗*^Pressure before kinking (mmHg)	^*∗*^Pressure after kinking (mmHg)	Pressure difference (mmHg)	
1	80	9.8	103.3	100.1		3.2
2	70	9.2	94.4	91.7		2.7
3	65	12.6	107.7	107.1		0.6
4	60	14.1	88.2	84.0		4.2
5	55	14.7	77.3	75.1		2.2
6	50	15.6	99.6	94.5		5.1
7	45	16.6	93.8	86.3	7.5	6.5
8	45	17.2	101.4	95.9	5.5	
9	30	27.2	89.7	80.0		9.7
10	25	25.8	94.6	83.3		11.3
11	15	27.5	99.7	84.2		15.5
12	10	26.9	106.0	85.0		21.0

^*∗*^Pressure: mean arterial blood pressure.
